# Disorder recognition in clinical texts using multi-label structured SVM

**DOI:** 10.1186/s12859-017-1476-4

**Published:** 2017-01-31

**Authors:** Wutao Lin, Donghong Ji, Yanan Lu

**Affiliations:** 1 School of Electronics Engineering and Computer Science, Peking University, Beijing, 100871 China; 2 School of Computer, Wuhan University, Wuhan, 430072 China

**Keywords:** Multi-label, Structured support vector machine, Information extraction, Clinical text

## Abstract

**Background:**

Information extraction in clinical texts enables medical workers to find out problems of patients faster as well as makes intelligent diagnosis possible in the future. There has been a lot of work about disorder mention recognition in clinical narratives. But recognition of some more complicated disorder mentions like overlapping ones is still an open issue. This paper proposes a multi-label structured Support Vector Machine (SVM) based method for disorder mention recognition. We present a multi-label scheme which could be used in complicated entity recognition tasks.

**Results:**

We performed three sets of experiments to evaluate our model. Our best F_1_-Score on the 2013 Conference and Labs of the Evaluation Forum data set is 0.7343. There are six types of labels in our multi-label scheme, all of which are represented by 24-bit binary numbers. The binary digits of each label contain information about different disorder mentions. Our multi-label method can recognize not only disorder mentions in the form of contiguous or discontiguous words but also mentions whose spans overlap with each other. The experiments indicate that our multi-label structured SVM model outperforms the condition random field (CRF) model for this disorder mention recognition task. The experiments show that our multi-label scheme surpasses the baseline. Especially for overlapping disorder mentions, the F_1_-Score of our multi-label scheme is 0.1428 higher than the baseline BIOHD1234 scheme.

**Conclusions:**

This multi-label structured SVM based approach is demonstrated to work well with this disorder recognition task. The novel multi-label scheme we presented is superior to the baseline and it can be used in other models to solve various types of complicated entity recognition tasks as well.

## Background

With the development of electronic records, analysis of clinical narratives becomes increasingly important since such narratives often contain vast quantity of useful information about patients and health [1].

In recent years, there has been a lot of work in information extraction from clinical texts. Earlier studies mainly focused on rule- or dictionary-based methods. As examples, MedLEE [2] used a vocabulary to recognize and classify words into semantic categories and then matched the sequences of semantic categories to structures defined in the grammar. MetaMap [3], which adopted a knowledge intensive approach, mapped biomedical texts to the UMLS [4, 5] Metathesaurus. Mork et al. [6] expanded a large number of term lists of drug phrases based on UMLS and used the lists to validate drug and indication relationships.

On the other hand, there have been various machine learning methods proposed recently for clinical text information extraction. Roberts et al. [7] treated a clinical relation extraction task which aims to extract relations between clinical entities such as a drug entity and a condition entity as a classification problem and applied Support Vector Machine (SVM) model to accomplish it. Lu et al. [8] considered chemical compound and drug recognition as a sequence labeling problem and developed a high-performance named entity recognition system by integrating Condition Random Field (CRF) with word clustering. He et al. [9] combined dictionary look-up and CRF method to recognize drug names. Zhu et al. [10] used SVM to separate biological terms frombiological non-biological terms, before they used CRF to determine the types of terms, which made full use of the power of SVM as a binary-class classifier and the data-labeling capacity of CRF.

**Fig. 1:**
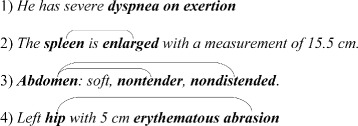
Examples of disorder mentions

In this paper, we present an approach to recognize disorder mentions from clinical narratives, which can be very complicated in some circumstances. In Fig. 1, sentence 1)–4) give some disorder mention examples.

In sentence 1), there is a disorder mention *dyspnea on exertion*, which is a contiguous disorder. In sentence 2), there is a disorder mention *spleen enlarged*, which is a discontiguous one. In sentence 3), there are two disorder mentions: *Abdomen nontender* and *Abdomen nondistended*. The two disorder mentions share the left boundary word *Abdomen*. In sentence 4), there are also two disorder mentions *hip abrasion* and *erythematous*. The span of the first disorder mention *hip abrasion* covers the second one *erythematous*. The disorder mentions in sentence 3) and 4) are overlapping ones.

Traditional structured SVM (SSVM) model [11] can recognize contiguous and discontiguous disorder mentions, but it has trouble recognizing overlapping disorder mentions. To accomplish this disorder mention recognition task, we describe a multi-label scheme, which can record the information of different disorder mentions in the overlapping cases at the same time. Combined with the multi-label scheme, our SSVM model performs well in the experiment.

## Related work

### Multi-label classification

There are two main strategies for multi-label classification: a) problem transformation methods and b) algorithm adaptation methods [12]. On the one hand, problem transformation methods transform multi-label problems into one or more single-label problems. Boutell et al. [13] solved the problem of semantic scene classification, where a natural scene may contain multiple objects such that the scene can be described by multiple class labels. They considered a multiple class label as a new single label. On the other hand, some classification algorithms can handle multi-label data directly, such as AdaBoost.MH and AdaBoost.MR [14], C4.5 algorithm [15] and ML-KNN [16].

### Complicated entity recognition

It is difficult to model entities that consist of discontiguous words or entities that share the same words. Based on BIO encoding scheme [17], Tang et al. [18] proposed the BIOHD multi-label method. In this method, “H” denotes head entities which are consecutive sequences of tokens shared by multiple disjoint concepts in a sentence while “D” denotes non-head entities which are consecutive sequences of tokens in a disjoint concept not shared by other disjoint concepts in a sentence. Later, Tang et al. [19] came up with a variant scheme BIOHD1234, where “1”, “2”, “3” and “4” indicate that a non-head entity is combined with the nearest head entity at left, the nearest non-head entity at left, the nearest head entity at right and the nearest non-head entity at right respectively. To recognize nested biomedical named entities, Lee et al. [20] came up with a two-phase method based on SVMs, which consists of a named entity boundary identification phase and a semantic classification phase.

### Overview of conference and labs of the evaluation forum 2013

The data set used in our work is from the task 1 of Conference and Labs of the Evaluation Forum (CLEF) 2013 (https://sites.google.com/site/shareclefehealth/). To the best of our knowledge, the best F_1_-Score for this data set is 0.783 so far, achieved by Tang et al. [19] in 2015.

## Methods

### Overall approach

We take the disorder mention recognition task as a sequence labeling problem. SSVM model performs well in classification tasks with complex outputs, such as trees, sequences, or sets [11], and we adopt the SSVM model to fulfill this task along with our multi-label scheme.

**Fig. 2:**
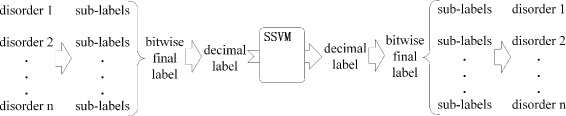
The flow of our model

The detailed algorithm flow is represented in Fig. 2. In the multi-label scheme, for every disorder mention that a token belongs to, there is a sub-label to record the disorder mention. All the sub-labels of each token would be integrated into just one bitwise multi-label, which is called a **final label**. Then we convert the final labels into decimal labels and feed the training data with decimal labels to our SSVM model. In the prediction phrase, what the SSVM model predicts are decimal labels as well, which will be converted into final labels. Finally, all the sub-labels will be extracted from final labels and the corresponding disoder mentions are obtained.

Since labeled data are always scarce while unlabeled data are abundant, we generate clustering-based word representations as features to reduce the dependence on the labeled data and further improve the model [21].

### Design of the multi-labels

As shown in Table 1, there are six types of **multi-labels**, *B*, *I*, *L*, *O*, *U* and *C*, all of which are in the form of 24-bit binary numbers. Labels in classes *B*, *I*, *L*, *O* and *U* stand for the Beginning, Inside, Last and Outside tokens of multi-token disorder mentions as well as Unit-length disorder mentions (those mentions made up of only one word), respectively, like the BILOU encoding scheme [22]. Labels in class *C* denote tokens that play different roles in several disorder mentions simultaneously. For example, a token, which is the beginning of a disorder and the last of another disorder at the same time, is represented by labels in class *C*.

**Table 1:** Design of the multi-labels

Type	Forms of the multi-labels
Class *U*	000000,000000,000000,*a*_6_ *a*_5_ *a*_4_ *a*_3_ *a*_2_ *a*_1_
Class *B*	000000,000000,*b*_6_ *b*_5_ *b*_4_ *b*_3_ *b*_2_ *b*_1_,000000
Class *L*	000000,*c*_6_ *c*_5_ *c*_4_ *c*_3_ *c*_2_ *c*_1_,000000,000000
Class *I*	*d*_6_ *d*_5_ *d*_4_ *d*_3_ *d*_2_ *d*_1_,000000,000000,000000
Class *C*	*d*_6_ *d*_5_ *d*_4_ *d*_3_ *d*_2_ *d*_1_,*c*_6_ *c*_5_ *c*_4_ *c*_3_ *c*_2_ *c*_1_,*b*_6_ *b*_5_ *b*_4_ *b*_3_ *b*_2_ *b*_1_,*a*_6_ *a*_5_ *a*_4_ *a*_3_ *a*_2_ *a*_1_
Class *O*	000000,000000,000000,000000

to make the 24-bit label easier to understand, extra commas are used to split the label

In Table 1, *a*_*i*_,*b*_*i*_,*c*_*i*_ and *d*_*i*_ (*i* = 1, 2, …, 6) represent binary 1 or 0; There are 6 variable bits (referred to as **variable region**) and 18 constant bits in labels in class *U*, *B*, *L* and *I*. The variable region lies in the rightmost 6 bits in labels in class *U*, the 7th to 12th bits from the right in labels in class *B*, the 13th to 18th bits from the right in labels in class *L* and the leftmost 6 bits in labels in class *I*. Meanwhile, the rest 18 constant bits of the above 4 types of multi-labels are filled with binary 0. Unlike the above 4 types of labels, labels in class *C* consist of 24 variable bits and labels in class *O* are made up of 24 bits of 0. Labels in class *C* can be divided into four variable regions, each of which has the same position with the variable region in labels in class *U*, *B*, *L* and *I*, respectively. Furthermore, some constraints need to be fulfilled in this multi-label scheme. There must be at least one bit 1 in labels in class *U*, *B*, *L* and *I*. And in labels in class *C*, there must be two or more variable regions where there is at least one bit 1. As for the **sub-labels**, except that there are no sub-labels in class *C*, the types of the sub-labels are the same as the multi-labels introduced above. Additionally, the sub-labels are made up of 1 bit binary 1 and 23 bits binary 0.

The reasons why we choose binary numbers as the multi-labels are as follows: 1) each bit stands for information of a disorder mention, so that a binary number, namely a multi-label, can record information of many disorder mentions. 2) bitwise operations make it convenient to integrate sub-labels into a final label and extract sub-labels from a final label.

### Application of the multi-labels

Although our multi-label scheme is based on BILOU scheme, the way we use it is different from traditional ways. In sentence 5), there is a disorder mention *tricuspid* *leaflets* *thickened*. In the traditional BILOU scheme, this sentence would be labeled as *The/O tricuspid/B valve/O leaflets/B are/O mildly/O thickened/B./O*. This method would run into trouble when there are multiple disorder mentions in a sentence. While in our method, this sentence would be labeled as *The/O tricuspid/B valve/O leaflets/I are/O mildly/O thickened/L./O*. There is therefore no confusion between multiple mentions and a single discontiguous mention using our method (when there may be more than six disorder mentions, we can expand the scope of the binary numbers).

5) *The* ***tricuspid*** *valve* ***leaflets*** *are mildly* ***thickened***.

6) ***Abdomen*** *is soft,* ***nontender***, ***nondistended*** *, negative* ***bruits***.

**Fig. 3:**
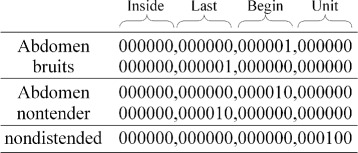
Examples of the sub-labels

The first step of this multi-label method is to assign each token sub-labels. Take sentence 6) as an example. There are three disorder mentions: *Abdomen bruits*, *Abdomen nontender*, and *nondistended*. When we implement our model, the disorder mentions are also encoded in this order, but actually the order of disorder mentions does not matter. The sub-labels of the three disorder mentions are shown in Fig. 3. In the beginning, we obtain the sub-labels of tokens through assigning 1 to the bits *a*_1_,*b*_1_,*c*_1_,*d*_1_ (referred to the bits of class *U*, *B*, *L* and *I* in Table 1) and 0 to other bits according to the token’s ordering in that disorder, just like sub-labels of the first disorder mention *Abdomen bruits*. We assign *Abdomen* a sub-label in class *B* with its bit *b*_1_ set to 1 and assign *bruits* a sub-label in class *L* with its bit *c*_1_ set to 1 since *Abdomen* is the beginning of the first disorder mention and *bruits* is the last. Then, if the next disorder mention overlaps with the former one, the sub-labels of the next disorder mention are acquired through assigning 1 to the bits *a*_2_,*b*_2_,*c*_2_,*d*_2_ and 0 to other bits, just like the sub-labels of the second disorder mention *Abdomen nontender* shown in Fig. 3. In the same way, when there are more disorder mentions overlapping with former ones, we obtain the sub-labels by assigning 1 to *a*_*i*_,*b*_*i*_,*c*_*i*_,*d*_*i*_, in which the subscript *i* increases one by one. Thus when the third disorder mention *nondistended* which overlaps with the former two comes, a sub-label in class *U* with its bit *a*_3_ set to 1 is assigned to the third mention *nondistended* since it is a unit-length disorder mention, as shown in Fig. 3. When there comes a disorder mention which does not overlap with any former disorder mentions within the sentence, the bits to be assigned 1 come back to *a*_1_,*b*_1_,*c*_1_ and *d*_1_. After that, we continue acquiring all the sub-labels by repeating the above process.

In view of the limited bits of our multi-labels, there can be up to six disorder mentions overlapping with each other. If needed, we can raise the limit by expanding the scope of the binary numbers that represent our multi-labels.

**Fig. 4:**
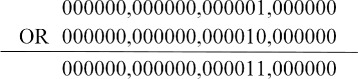
Example of Algorithm 1

After all the sub-labels of every token are obtained, we need to integrate them into a final label by doing bitwise OR operation, as the Algorithm 1 shows. Take the token *Abdomen* in sentence 6) as an example, as shown in Fig. 4, its sub-label “ 000000,000000,000001,000000” and “ 000000,000000,000010,000000” are integrated into a final label “ 000000,000000,000011,000000”. Every binary 1 in the final label indicates the information of a disorder mention. For example, the final label “ 000000,000000,000101,000000” means its corresponding token is the beginning of two different disorder mentions; another example of the final label “ 000010,000001,000000,000000” means this token is not only an inside token of a disorder mention but also the last token of another mention. The final labels of tokens of sentence 6) are listed in Table 2.

**Table 2:** Examples of the final labels

Token	Final label
Abdomen	000000,000000,000011,000000
is	000000,000000,000000,000000
soft	000000,000000,000000,000000
,	000000,000000,000000,000000
nontender	000000,000010,000000,000000
,	000000,000000,000000,000000
nondistended	000000,000000,000000,000100
,	000000,000000,000000,000000
negative	000000,000000,000000,000000
bruits	000000,000001,000000,000000



**Fig. 5:**
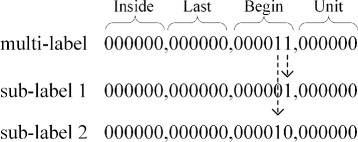
Example of Algorithm 2



When prediction is finished and the predicted decimal labels have been converted into bitwise final labels, the next step is to extract sub-labels from final labels using Algorithm 2. The AND operator denotes the bitwise AND operation. Algorithm 2 scans all the 24 bits in a final label and it will output a sub-label for each bit 1 in the final label. Take the token *Abdomen* in sentence 6) as an example again, as shown in Fig. 5, there are two bits 1 in its final label “ 000000,000000,000011,000000”, which lie in the 7th and 8th bits from the right. Correspondingly, Algorithm 2 will output two sub-labels “ 000000,000000,000010,00000” and “ 000000,000000,000001,00000”, whose 7th and 8th bit from the right are assigned bit 1 respectively. After all the sub-labels are extracted, we need to gather sub-labels which have the same ranking position of binary 1 in their variable region and then extract the disorder mentions as BILOU encoding scheme does. For instance, the sub-label sequence (sub-label in class *B* with its bit *b*_2_ set to 1, sub-label in class *I* with its bit *d*_2_ set to 1, sub-label in class *L* with its bit *c*_2_ set to 1) can be regarded as a label sequence (*B*, *I*, L) in the BILOU scheme.

Combining the SSVM model with this multi-label scheme, we can not only deal with the contiguous and discontiguous disorder mentions, but also the overlapping ones.

### Feature generation

We exploit several types of features:

*(1) General linguistic features.* These include the classic features for named entity recognition tasks, such as Bag of Words (BOW) and Part of Speeches (POS). Tokenization and POS tagging are conducted by Stanford CoreNLP toolkit [23].

*(2) Capitalization features.* The reason why we use capitalization features is that various spelling habits of different people lead to different spellings of the same word. For instance, some doctors tend to write three times a day as “t.i.d.” while others may write “T.I.D.” instead. Moreover, the grammar rule that the first word of a sentence should begin with a capital letter while the same word in other position should not is also a reason for that.

*(3) Case pattern features.* Case pattern features are helpful since mentions of the same semantic type often have similar capitalization patterns, such as C-polyp (Cervical Polyps) and E-polyp (Endometrial Polyps).

*(4) Word representation features.* Previous studies showed that the unsupervised word representation features are beneficial to clinical named entity recognition tasks [21]. A common approach to induce word representation is to use clustering [24]. The unlabeled texts used for word clustering are the discharge records and various medical examination reports of 31,507 patients derived from MIMIC (Multiparameter Intelligent Monitoring in Intensive Care) II Databases (http://physionet.org/mimic2/). Word clustering is conducted by word2vec [25, 26], which provides an efficient implementation of the continuous bag-of-words and architectures for computing vector representations of word.

*(5) Contextual features.* For each token, we combine above features of the contextual tokens together as the contextual features.

The detailed feature descriptions are presented in Table 3.

**Table 3:** Feature set description

Feature	Description
Bag of Words	Bag of Words in a 5-word window.
Part of Speeches	Part of Speeches in a 7-word window.
Capitalization	Convert all alphabetic characters of the words to uppercase [31]. The window size is 5.
Case pattern	The patterns are generated by the following steps. Similar to [32], any uppercase alphabetic character is replaced by “A” and any lowercase one is replaced by “a”. In the same way, any number is replaced by “0”. The window size is 3.
Word representation	We use word2vec to acquire 700 clusters from the unlabeled clinical narratives and give each cluster a different serial number. Then we take the serial number of the clusters as a feature. The window size is 3.

### Experiments

#### Data set

The data set we used comes from task 1 of CLEF 2013. There are 199 clinical reports in the training set and 99 clinical reports in the test set. The clinical reports include discharge records, electrocardiogram, echocardiogram and radiology reports. Table 4 gives the statistics for the three types of disorder mentions: contiguous and non-overlapping (referred to as **contiguous**), discontiguous and non-overlapping (referred to as **discontiguous**), and **overlapping**. When multiple discontiguous disorder mentions overlap with each other, these mentions are categorized as overlapping.

**Table 4:** Statistics for three types of disorder mentions

Disorder type	Amount	Percentage
Contiguous	9867	88.45%
Discontiguous	565	5.06%
Overlapping	724	6.49%
Total	11156	100.00%

Table 5 shows the statistics for different types of discontiguous disorder mentions(including 524 overlapping disorder mentions which are in the discontiguous form). A **breakpoint** refers to consecutive tokens that separate a disorder mention. For instance, in sentence 5), there are 2 breakpoints in the disorder mention *tricuspid leaflets thickened*. The disorder mentions in the data set have two breakpoints at most.

**Table 5:** Statistics for discontiguous disorder mentions

Disorder type	Amount	Percentage
1 breakpoint	1027	94.31%
2 breakpoints	62	5.69%
3 or more breakpoints	0	0.00%
Total	1089	100.00%

Table 6 presents the statistics for overlapping disorder mentions. In the first column, the number means how many disorder mentions overlap with each other at the same time. As an example, there are 3 disorder mentions: *Abdomen bruits*, *Abdomen nontender* and *nondistended*, which overlap with each other in sentence 6). According to the statistics, there can be up to 6 disorder mentions overlapping with each other at the same time.

**Table 6:** Statistics for overlapping disorder mentions

Disorder type	Amount	Percentage
2 disorder mentions overlap with each other	482	66.57%
3 disorder mentions overlap with each other	198	27.35%
4 disorder mentions overlap with each other	28	3.87%
5 disorder mentions overlap with each other	10	1.38%
6 disorder mentions overlap with each other	6	0.83%
7 or more disorder mentions overlap with each other	0	0.00%
Total	724	100.00%

Table 7 gives the statistics of disorder mentions with different span lengths. The **span length** means the distance between the first and last token of a disorder mention. For example, in sentence 5), the span length of the disorder mention *tricuspid leaflets thickened* is 6 since the distance between *tricuspid* and *thickened* is 6. Specially, the span length of a unit-length disorder is 1.

**Table 7:** Disorder mentions with different span lengths

Span length	Disorder amount	Percentage
1	5172	46.36%
2	3158	28.31%
3	1580	14.16%
4	474	4.25%
5	340	3.05%
6 or more	432	3.87%
Total	11156	100.00%

Among all the disorder mentions in the testing data set, the percentage of new disorder mentions, namely mentions that do not appear in the training data set, is about 40.72%.

#### Evaluation metrics

We use the precision, recall and F_1_-Score in (1)-(3) to evaluate the performance [27].

1 $$  Precision = \frac{TP}{TP + FP}  $$

2 $$  Recall = \frac{TP}{TP + FN}  $$

3 $$  F_{1}\textrm{-}Score = \frac{2 * Precision * Recall}{Precision + Recall}  $$

Two evaluation modes are adopted. The strict mode requires that the predicted spans should be exactly the same as the answer. Relaxed mode includes left match and right match mode. Left match means the prediction is judged as correct as long as the left boundary matches correctly and right match is judged by the right boundary [28]. All the results presented below are evaluated in strict mode, unless explicitly specified.

#### Experimental setup

We designed the following experiments to evaluate our model. First, in order to show the effect of the features we described above separately, a series of controlled experiments were set up. In these experiments, we added the features to the feature set one by one. Second, CRF model is widely used in sequence labeling tasks, therefore we take CRF model as a baseline to compare with our SSVM model. The features and the multi-labels employed in the CRF model are exactly the same as those in our SSVM model. Last, in order to show the performance of our multi-label scheme, SSVM model with the BIOHD and BIOHD1234 scheme, with which Tang et al. [19] achieved the best F_1_-Score so far, are adopted as a baseline. The features employed are exactly the same as those in our SSVM model. We trained SSVM models with SVM-HMM [29] and CRF model with CRF++ [30]. The parameters of our SSVM model and baseline models were optimized by 10-fold cross-validation on the training data set.

## Results and discussion

### Overall performance

Table 8 gives the results for the multi-label SSVM model with different feature sets. From the results we can see that the features, e.g., capitalization and word representation features, mainly improve the recall. In particular, an SSVM model with word representation features can recognize about 19.34% more correct new disorder mentions than SSVM model without word representation features. Sentence 7) and sentence 8) are two examples.

7) *Past Medical History:* ***Hypertension***. ***Addison’s disease***. ***Hypothyroidism***. ***Melanoma***. ***BPH***.

**Table 8:** Results for multi-label SSVM model with different feature sets

Feature set	Precision	Recall	F _1_-Score
SSVM + BOW	0.7626	0.3329	0.4635
SSVM + BOW + POS	0.7953	0.3857	0.5195
SSVM + BOW + POS + capitalization	0.8417	0.5702	0.6799
SSVM + BOW + POS + capitalization + case pattern	0.8398	0.5839	0.6889
SSVM + BOW + POS + capitalization + case pattern + word representation	0.8244	0.6620	0.7343

8) Upon arrival to [** Hospital1 2 **] in preparation for cath, patient noted to be ***thrombocytopenic*** *to 140.*

In sentence 7), there are five disorder mentions, *Hypertension*, *Addison’s disease*, *Hypothyroidism*, *Melanoma* and *BPH*. Our model could not recognize *Melanoma* and *BPH* until we added the word representation features. Likewise, word representation features enable our model to recognize the disorder *thrombocytopenic* in sentence 8).

### Results in different evaluation modes

Table 9 shows the results for the model in different evaluation modes. It indicates that the performance for the right match outperforms that for left match. To explore the reasons for the difference, we consider the following cases in detail.

**Table 9:** Results for different evaluation modes

Mode	Precision	Recall	F _1_-Score
Strict	0.8244	0.6620	0.7343
Relaxed (left match)	0.8229	0.6826	0.7462
Relaxed (right match)	0.8441	0.6995	0.7650

There are many mistakes in recognizing left boundary of contiguous disorder mentions. In some cases, adjectives and nouns before disorder mentions are misjudged as the beginning of the mention. For example, there is a disorder mention *fluid collects* in sentence 9), while the prediction of the model is *abdominal wall fluid collects*. In some other cases, adjectives and nouns located at the left boundary of contiguous disorder mentions are often omitted. In sentence 10), there is a disorder mention *Multiple renal cysts*, while the prediction is *renal cysts*.

9) *Reason: please drain abdominal wall* ***fluid collects*** *(x 2) with ultras.*

10) ***Multiple renal cysts***.

As we can see from Table 4, contiguous disorder mentions account for 88.45% of all the mentions. Furthermore, 23.59% of contiguous disorder mentions appear after adjectives or nouns and the first tokens of 92.86% of contiguous disorder mentions are adjectives or nouns so that this type of mistake makes a great difference.

### Performance for different types of disorder mentions

Table 10 shows the performance for contiguous, discontiguous and overlapping disorder mentions respectively.

**Table 10:** Results for different types of disorder mentions

Type	Item	Value
Contiguous	Precision	0.8262
	Recall	0.7036
	F _1_-Score	0.7600
Discontiguous	Precision	0.6914
	Recall	0.3060
	F _1_-Score	0.4242
Overlapping	Precision	0.8632
	Recall	0.2832
	F _1_-Score	0.4265

#### (1) Contiguous disorder mentions

Our model obtains the highest performance in recognizing contiguous disorder mentions among these three types of mentions. It is clear from the data that contiguous mentions are easier to recognize than the other two types of mentions. Additionally, about 57.33% of contiguous disorder mentions in our testing data are unit-length mentions, which are in the simplest form of disorder mentions.

#### (2) Discontiguous disorder mentions

The results show that the recall of discontiguous disorder mentions is not good enough. The reason for that are: a) The samples of discontiguous disorder mentionss are too few, which only account for 5.06% of all the disorder mentions. b) As Table 7 shows, in some cases, the span lengths of many discontiguous disorder mentions are too large so that our model cannot capture their features.

#### (3) Overlapping disorder mentions

The weakness of recognizing overlapping disorder mentions lies in the recall as well. There are mainly two reasons.

a) The samples of overlapping disorder mentions only account for 6.49%. What’s more, Table 6 indicates that the more disorder mentions overlap with each other at the same time, the sparser the multi-label of them will be. Thus the performance in predicting tokens whose label contains many bits 1, namely the token belongs to many disorder mentions, is poor. But from another perspective, tokens which belong to many disorder mentions simultaneously are rare so that they will not affect the final result too much. When the percentage of overlapping disorder mentions rises, the result would be better.

b) A disadvantage of our multi-label scheme is that the multi-labels of the same disorder mention may be different in some situations. Sentence 11) and 12) are two examples (these two examples are simplified versions of the original sentences, because there are too many disorder mentions in the original ones).

11) ***Abdomen***: ***nontender***.

12) ***Abdomen***: ***nontender***, ***nondistended***.

In sentence 11), there is only one disorder mention *Abdomen nontender*. According to the multi-label scheme, the bit *b*_1_ of the multi-label of *Abdomen* and the bit *c*_1_ of the multi-label of *nontender* would be assigned 1 because *Abdomen* is the first and *nontender* is the last token of disorder mention *Abdomen nontender*. Thus, the label of *Abdomen* is “ 000000,000000,000001,000000” and the label of *nontender* is “ 000000,000001,000000,000000”. But in sentence 12), there are two disorder mentions *Abdomen nondistended* and *Abdomen nontender*. The bits *b*_1_ and *b*_2_ of the label of *Abdomen* would be assigned 1 because it is the beginning of both the two disorder mentions; the bit *c*_1_ of the label of *nondistended* would be assigned 1 because it is the last token of the first disorder mention *Abdomen nondistended*; the bit *c*_2_ of the label of *nontender* would be assigned 1 as well because it is the last token of the second disorder mention *Abdomen nontender*. Thus, the label of *Abdomen*, *nontender* and *nondistended* are “ 000000,000000,000011,000000”, “ 000000,000010,000000,000000” and “000000,000001, 000000,000000”, respectively. Therefore, the same disorder *Abdomen nontender* may have different multi-labels in different situations so that our model may be confused. Since the situation of sentence 11) would occurs much more frequent than sentence 12), our model would tend to predict the label of the disorder mention *Abdomen nontender* as in sentence 11). To some extent, this characteristic weakens the performance of our model.

### Comparison with baselines

#### Baseline 1: CRF model with our multi-label scheme

**Fig. 6:**
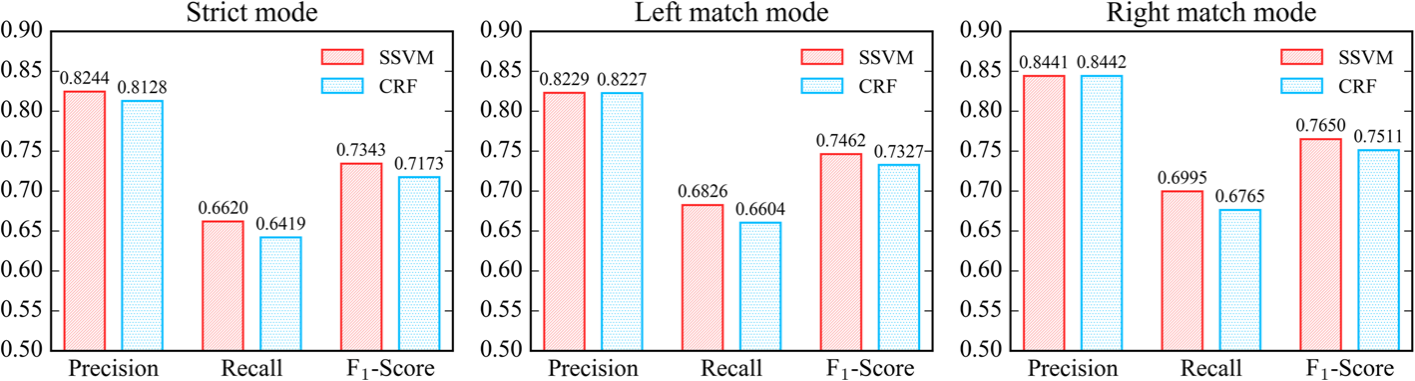
Comparison between SSVM and CRF model

As shown in Fig. 6, in strict mode, the best F _1_-Score of CRF model is 0.7173 while the best F _1_-Score of our SSVM model is 0.7343; in left match mode, the best F _1_-Score of CRF model is 0.7327 while the best F _1_-Score of our SSVM model is 0.7462; in right match mode, the best F _1_-Score of CRF model is 0.7511 while the best F _1_-Score of our SSVM model is 0.7650. Therefore, we can see SSVM model outperforms CRF model in this task.

#### Baseline 2: SSVM model with BIOHD and BIOHD1234 scheme

**Fig. 7:**
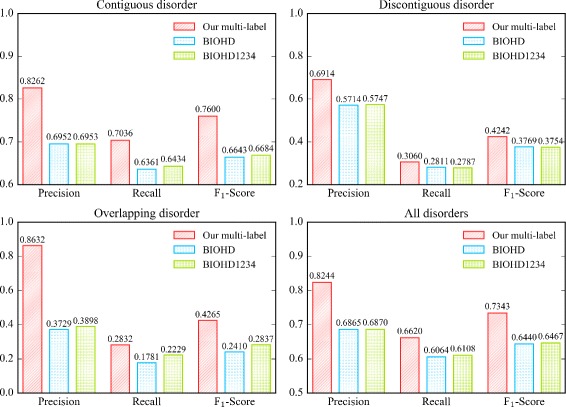
Comparison among BIOHD, BIOHD1234 and our multi-label scheme

BIOHD and BIOHD1234 multi-label scheme can deal with discontiguous and overlapping disorder mentions. But they also have some limitations. As Tang said in [19], neither BIOHD nor BIOHD1234 scheme can represent sentences which contains two or more head entities, such as sentence 13) where there are two disorder mentions *blood third ventricles* and *blood four ventricles*. There are other complicated situations that neither BIOHD nor BIOHD1234 can deal with, such as sentence 14) where there are two disorder mentions *atrial pacemaker artifact* and *pacemaker capture*. However, our multi-label scheme can handle all these complicated situations. Figure 7 shows the results of BIOHD, BIOHD1234 and our multi-label scheme. The performance for contiguous, discontiguous and overlapping disorder mentions are showed respectively.

13) *There is a small amount of* ***blood*** *seen within the* ***third*** *and* ***fourth ventricles***.

14) *There is intermittent appearance of apparent* ***atrial pacemaker artifact*** *without* ***capture***.

For all the three types of disorder mentions, the F _1_-Scores of our multi-label scheme are higher than BIOHD and BIOHD1234. In particular, for overlapping disorder mentions, the F _1_-Score of our multi-label scheme is 0.4265 while the score of BIOHD and BIOHD1234 are only 0.2410 and 0.2837 respectively. Because there are few complicated sentences like sentence 13) and 14) in the data set, the advantage of our multi-label scheme is not fully reflected. In addition, when the percentage of overlapping disorder mentions rises, the performance of our model in recognizing overlapping disorder mentions would be better.

The experiments demonstrate that our multi-label scheme is better than Tang’s BIOHD and BIOHD1234 in this task. To figure out why our total F _1_-Score does not catch up with Tang’s best F _1_-Score 0.783, we performed a baseline in which we removed all discontiguous and overlapping disorder mentions, then we trained an SSVM model with BIO scheme and our features, so that we can compare it with the results got under the same conditions except the feature sets in [19]. The results showed in Table 11 indicate that features used by Tang et al. [19] are more effective than ours. This might be the reason why Tang’s F _1_-Score is higher than ours. We would like to try more features to boost the performance of our model in our future work.

**Table 11:** Comparison between SSVM model with different feature sets

Features	Precision	Recall	F _1_-Score
Our features	0.6560	0.5875	0.6199
Tang’s features	0.842	0.722	0.777

### Error analysis

With further analysis, the main errors of our model are categorized as follows.

#### (1) New disorder mention prediction error

The new disorder mentions account for 40.72% in average among all the mentions in testing data set. Although we added word representation features to increase the model’s ability to recognize new disorder mentions so that the recall of those mentions increased from 0.4565 to 0.6498, there are still 36.02% of new mentions not recognized. The possible reasons are: a) some of the new disorder mentions have a very long span length (this will be explained in the following part *Long distance dependency*). b) some of new disorder mentions have a complex structure so that there are few disorder mentions have similar features with them in the training data. Consider the disorder mention *elevated CE’s* in sentence 15) as an example, few disorder mentions have the similar case pattern feature *aaaaaaaa AA’a*, POS feature *JJ NN POS* and capitalization feature *ELEVATED CE’S* in the context.

15) *He was noted to have* ***ST segment elevations*** *in inferolateral leads,* ***elevated CE’s***

#### (2) Boundary error

Errors often occur in the boundary of a disorder. a) Adjectives and nouns before contiguous disorder mentions are sometimes misjudged as the beginning of the disorder. The examples in sentence 9) demonstrate these situations. b) adjectives and nouns located at the left boundary of contiguous disorder mentions are often omitted. The examples in sentence 10) demonstrate these situations.

#### (3) Long distance dependency

As Table 7 shows, the span length of some disorder mentions in the data are pretty long. For instance, the span length of the disorder Abdomen tenderness in sentence 16) reaches 16. Long span length increases the difficulty of disorder recognition, especially when the span length exceeds the feature window sizes.

16) ***Abdomen*** *: soft,* ***NT/ND*** *, normoactive BS, no* ***masses*** *, no* ***rebound*** *or* ***tenderness***.

**Fig. 8:**
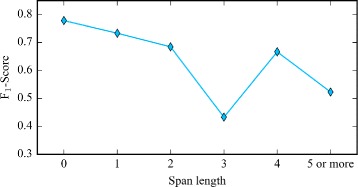
Results for disorder mentions with different span lengths

Figure 8 shows the result for recognizing disorder mentions with different span lengths. The blue broken line indicates the F _1_-Scores. Figure 8 illustrates that as the span length increases, the performance descends. Therefore, our model fails to recognize many disorder mentions because their span lengths are too long and we do not capture complex features.

### Summarization for the multi-label scheme

To summarize, the major advantage of our multi-label scheme is that it can handle complicated situations in entity recognition tasks. The major limitations of our scheme are: 1) because the multi-labels of the same disorder mention may be different in some situations, the training instances of complicated disorder mentions would be sparse. 2) The number of possible disorder mentions is limited by the bits used in the multi-label scheme. However, the situations where there are more than six entities in a sentence are rare. Moreover, we can use more bits to raise the limit if needed.

## Conclusions

Aiming at the disorder recognition task,we integrate a multi-label version of the BILOU scheme with an SSVM model to create a novel multi-label SSVM model. Using binary digits to record the disorder mention details, the multi-label scheme enables us to recognize complicated disorder mentions, e.g., those overlapping with each other. The best F _1_-Score of our model is 0.7343. In addition, for overlapping disorder mentions, the F _1_-Score of our multi-label scheme is 0.1428 higher than the baseline “BIOHD1234” scheme. This shows the perspective of the multi-label scheme in dealing with recognition of complicated named entities in biomedical text mining.

In the future, we would like to generate more features such as semantic group features. We also intend to address the problems described in the section Error Analysis. Furthermore, we would like to try some other models such as neural network to recognize disorder mentions from clinical texts.

## Abbreviations

BOW: Bag of words
CLEF: Conference and labs of the evaluation forum
CRF: Condition random field
MIMIC: Multiparameter Intelligent Monitoring in Intensive Care
POS: Part of speeches
SSVM: Structured support vector machine
SVM: Support vector machine
